# Novel Method of Entering Bladder

**Published:** 1886-03

**Authors:** 


### CULLINGS FROM CONTEMPORARIES.

### NOVEL METHOD OF ENTERING THE BLADDER WHERE ALL OTHER MEANS FAIL.

DR. WILLIS P. KING, of Sedalia, Mo., gives (*Courier of Medicine*, March, 1886) an account of how he first succeeded in introducing a catheter into a child, after repeated efforts to enter the bladder by other means. This case was not a stricture, but simply obstruction to the urethra by the pressure of an abscess in the perineum. Recently he had a very bad case of permanent stricture, where there was total occlusion of the urethra, and when all means failed to pass it, even to the filliform bougie, and external urethrotomy was apparently inevitable, the Doctor thought of his first case, and proceeded to adopt the same principle, though in a different manner; in the child's case the Doctor used his mouth for the fountain syringe, and received the urine in his eye; in the latter, he used a syringe and caught the urine in a vessel. Introducing a silver catheter as far as it would go, and pressing the urethra tightly to it, he filled a syringe, with a conical nozzle, with warm water, and, introducing the nozzle into the catheter, forced the water swiftly out. Afer doing this two or three times, and finding that part of it, at least, went through, he pressed the catheter in, and, to his surprise and gratification, it entered the bladder. After which he passed a Goulay's divulsor and divulsed the stricture; getting in next a No. 16, and then a No. 18 (Am. Size) bougie.

We suggest to Dr. W., if he is ever similarly situated, and his method fails, which it must do in some old tough narrow and tortuous stricture, to try the method which served us successfully in an emergency, a small—the smallest size—fiddle string. It will, if softened at the end and oiled, pass through the narrowest and most tortuous canal (where a bristle will go), and more over, swelling with moisture, it dilates the stricture; next introduce the largest size fiddle string. These expedients are well worth remembering by isolated practitioners, for they never know when life may depend upon a knowledge of such resources.

